# Wedl's Pathology of the Teeth

**Published:** 1873-04

**Authors:** 


*Wedl's Pathology of the Teeth.*—Some complaint having been expressed in a prominent Dental Journal concerning this work, as containing so much that is new—a result of the minute investigations of the author—that it is beyond the comprehension of most students, and proves unsatisfactory except to the more advanced members of the dental profession, the "*Boston Medical and Surgical Journal*" regrets that such an opinion should be entertained, in the following appropriate language:

"We cannot fail to express our regret that such an opinion should be entertained by so discriminating a professional organ. We feel sure that the statement is disproved in our own neighborhood at least, where dental students have long waited for just such a thorough scientific work as that which Prof. Wedl has offered; where the study of general human pathology has kept pace during the past few years with the most advanced investigations of foreign savans; and where instructors in the dental as well as the medical schools are *determined* that the best, the most thorough and scientific training shall be afforded their students, as well as that which is more apparently practical and useful.

"If the sole object of dental education is to teach men how to extract and fill teeth, then such a volume as the one in question is indeed out of place; but we are glad to recognize the fact that dentistry of the present day has risen above the plane which makes it simply an art, and that it has become a science. It must keep pace with medicine, recognizing the facts in pathology which are now universally and unreservedly accepted, or it will cease to maintain the respect we have learned to entertain for it. We cannot help feeling that such views are behind the day, that they are unwarranted in fact, and unworthy to be advanced by dental educators."

